# A Narrative Review of the Carcinogenic Effect of Night Shift and the Potential Protective Role of Melatonin

**DOI:** 10.7759/cureus.43326

**Published:** 2023-08-11

**Authors:** Elvina C Lingas

**Affiliations:** 1 Hospital Medicine, Presbyterian Hospital, Albuquerque, USA

**Keywords:** oncology, high-risk breast cancer, preventive oncology, occupational hazards, breast cancer prevention, disability & cancer prevention, nurses, nocturnist, night shift, cancer risk

## Abstract

Since the IARC (International Agency for Research on Cancer) announcement in 2007 indicating the possibility of night-shift work carrying carcinogenesis risk, multiple studies on a global level have been conducted to investigate the correlation between night-shift work and cancer development. Circadian rhythm disruption and decreased melatonin production have been postulated as potential contributing factors. There is also growing evidence that night-shift workers tend to adopt unhealthier lifestyles which contribute to poorer health and increase the risk of developing diseases such as cancer. No experimental study has been specifically dedicated to testing specific methods that could decrease cancer risk in night-shift workers. While there are a few studies that investigate melatonin's concurrent use with chemotherapy in cancer patients, there is yet to be seen for studies that investigate melatonin specifically as a cancer prevention method. This narrative review aims to examine current evidence of healthcare night-shift work’s risk in cancer incidence, potential pathogenesis, and its significance in clinical practice.

## Introduction and background

Night shifts are getting increasingly common in the modern world as 24-hour service expectation is present in day-to-day sectors such as hospitals, hospitality, transportation, and security [1]. In 2007, IARC (International Agency for Research on Cancer) concluded that night shift work is a probable carcinogen to humans based on animal and epidemiological studies [2]. The contributing factors for this are hypothesized to be directly related to circadian rhythm and decreased melatonin production which are thought to be oncogenic in nature [3,4]. Melatonin, secreted mostly in the pineal gland, is involved in multiple cellular processes including cell cycle regulation, cell proliferation, cell survival, apoptosis, DNA repair, and tumor suppression [5]. Melatonin seems to also be related to modulation and gene expression in estrogen which is important in breast cancer [5-7].

Since 2007 to date, we have had multiple studies suggesting a potential carcinogenic effect of night shift work. Most published studies investigated the relationship between night-shift work and breast cancer risk. Schernhammer et al. conducted 10-year prospective cohort studies among nurses who worked night shifts, and nurses who have worked more than 30 years of night shifts are 36% more likely to have breast cancer compared to the ones who had not worked night shifts before [8]. Another cohort study conducted by the same author reflected a similar result [9]. Two systematic reviews examining night-shift workers suggest increasing evidence of association with increasing cancer incidence and all-cause mortality due to disruption of circadian rhythm and melatonin suppression [10,11]. A recent meta-analysis by Wei et al. showed increased risk of developing breast cancer in night-shift workers who have worked for more than 10 years as well as an increase in cardiovascular mortality, which mirrored previous meta-analysis by the same author that not only showed increased risk of developing breast cancer and increase in mortality but also showed increased risk of breast cancer morbidity [12,13]. Another study indicated weak evidence and less strong association [14]. Potential mechanisms include disruption of circadian rhythm and decreased melatonin which has anti-cancer properties. There have been few studies that investigate the relationship between night-shift work and other types of cancer such as lung cancer [15], bladder cancer [16], prostate cancer [17], and esophageal cancer [18].

The objective of this narrative review is to summarize the carcinogenic effect of night-shift work with an emphasis on healthcare workers and the potential protective role of melatonin in cancer risk, with a focus on breast cancer.

Literature search method

The search was performed to include articles from PubMed and Google Scholar that investigate night-shift risk and cancer between 2000 and 2022. Original articles and review articles are both included. Articles in foreign languages besides English were excluded. 

## Review

Disruption of circadian rhythm and role of melatonin in cancer development including breast cancer

Circadian rhythm is an internal biological process that regulates mechanisms such as metabolism, DNA repair, and immune system regulation which are all important parts of cancer pathogenesis. Working circadian rhythm enables human bodies to function for 24 hours around the clock [19]. Circadian rhythm is regulated by feedback loops of the circadian genes located in the suprachiasmatic nuclei as well as in the peripheral tissues. Disruption in circadian rhythm has been observed in vivo as the precursor of failure in managing cell proliferation and apoptosis regulation. There are eight core genes of circadian rhythm identified, which have their own functions respectively [20]. Experimental studies in animals have shown a possible carcinogenesis effect of light work exposure during night time [2], and other animal studies have shown that mice with inability to regulate circadian rhythm effectively have increased cancer risk [21,22]. Disruption in circadian rhythm also may increase progression of already existing cancer. Rats with sarcoma have increased tumor growth when kept in the alternative light/dark cycle compared to the constant cycle [23]. A study by Bollati et al. where they examined peripheral blood from male shift workers showed night shift potentially has induced DNA methylation defect including changes in Alu repetitive elements methylation and gene-specific methylation of inflammatory genes such as IFN-γ and TNF-α [24].

**Figure 1:**
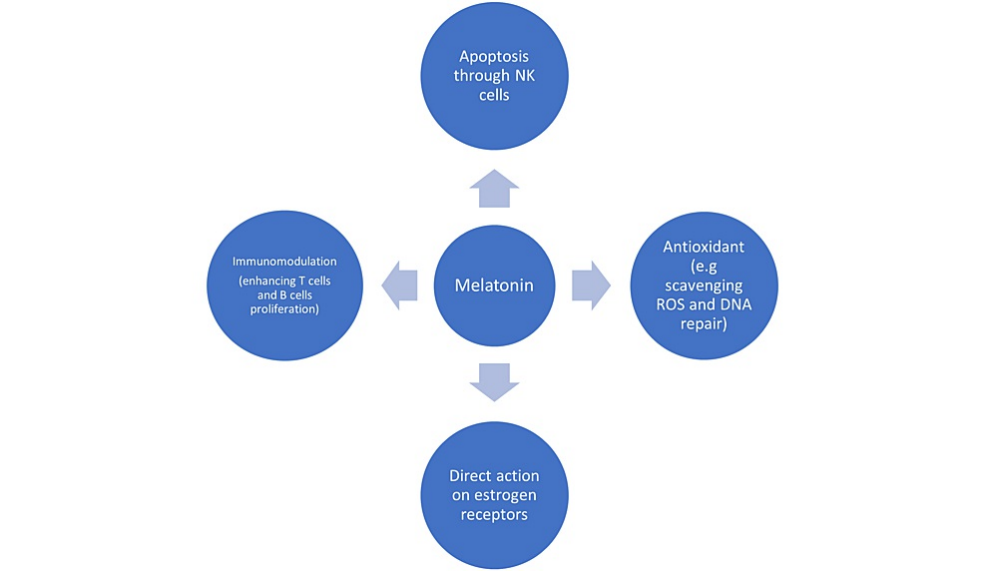
Melatonin's Anti-cancer Properties Credit: Author Elvina Lingas NK: Natural killer; ROS: reactive oxygen species; DNA: deoxyribonucleic acid

Melatonin is mainly secreted in the pineal gland and other peripheral organs [25,26] whose functions ranged from circadian rhythm monitoring, immunomodulation, anti-inflammation enhancement, and vasoregulation [27,28]. Melatonin’s anti-oncogenic properties have been experimented on animals and its anti-carcinogenesis properties are due to its antioxidant, immunomodulation, and apoptotic regulatory functions. Melatonin can protect against DNA damage either by scavenging reactive oxygen species or by stimulating the DNA repair system. [25] Melatonin influenced NK (natural killer) cells which are responsible for destroying cancer-affected cells [28]. Melatonin also inhibits the AKT/mTOR pathway and ERCC1 that induces autophagy and increases apoptosis of cancer cells [29]. There is in vivo evidence that melatonin enhances the function of mature NK cells by increasing T helper 2 cells’ production of IL-2 (interleukin-2) [30]. Melatonin also enhances T lymphocyte cell proliferation and increases antigen-presenting cells as well as having an anti-apoptosis effect on improving the survival of normal granulocytes and B lymphocytes [28]. In a mice model injected by tumor cells, mice that were administered melatonin survived beyond three months [31]. In addition to direct oncostatic action and immunomodulatory properties, in animal model studies, melatonin has been shown to prevent nitrosodiethylamine (NDEA)-induced liver cancer. Melatonin also inhibits the growth of prostate tumors via activation of MT1 receptors thereby inducing translocation of the androgen receptor to the cytoplasm and inhibition of the effect of endogenous androgens [32]. In estrogen receptor alpha (ERα)-positive human breast cancer, melatonin, via the MT1 receptor, suppresses ERα mRNA expression and ERα transcriptional activity [5]. Melatonin is also found to have SERM (selective estrogen receptor modulators) and SEEM (selective estrogen enzyme modulators) properties thus potentially can be used as adjuvant therapy for estrogen-positive breast cancer [33]. Moreover, it seems to also be capable of inhibiting angiogenesis, cancer cell invasion, and telomerase activity [34]. Melatonin also downregulates VEGF, EGF, and endothelin-1 gene production to further inhibit angiogenesis [29]. It also upregulates the p53/MDM2 pathway that has been studied for novel cancer treatment [29,35]. Figure 1 shows melatonin’s anti-cancer mechanism.

Night shift and risk of developing an unhealthy lifestyle

Night shift has been documented to also increase the risk of metabolic disorders such as type II diabetes mellitus (DM). This study showed that coupled with a night shift unhealthy lifestyles such as smoking, being sedentary, and being overweight have an additional 11% risk of developing type 2 DM [36]. A review by Pepłońska et al. showed that nurses who do night-shift work tend to have lower fruit and vegetable consumption [37]. A questionnaire-based observational study by the same author showed that night-shift workers also tend to be more sedentary, although of note alcohol consumption tends to be lower [38]. We have sufficient evidence over the years regarding the lifestyle changes that affect cancer. Diets rich in fruits and vegetables can yield anti-cancer properties due to their abundant antioxidants [39]. Obesity and a sedentary lifestyle have been linked to an increased risk of cancer [40]. This cross-sectional study of 2059 female nurses showed that there is a positive association between night shift and having higher BMI (BMI >25) [41]. In recent studies, physical activity increased the methylation activity of LINE1 genes [42], and the methylation activity of these genes is associated with chromosomal instability and inflammation [43].

Some evidence suggested that night-shift workers may have an increased rate of tobacco smoking [44], and night-shift nurses in these population-based mortality data are shown to have increased risk of lung cancer even after the smoking risk is adjusted [45]. The Korean study from National Survey data of smoking prevalence in men and women with various work types and socioeconomic status showed that adjusted ORs were elevated in night-shift workers but they were not statistically significant [46]. The Swedish study of 197,653 workers in contrast showed that night-shift workers are more likely to smoke [47]. In a study of two large cohorts of night-shift workers, it was shown that night-shift workers are more likely to have higher BMI and current smokers [36].

Night-shift work and cancer in clinical practice

Night-Shift Work and Breast Cancer

Most published studies that investigated the correlation between night-shift work and cancer risk had focused on specifically breast cancer risk. The National Toxicology Program performed hazard analysis from multiple studies and indicated that there is a strong association between night-shift work and breast cancer risk [48]. Those studies’ inclusion ranged from rotating night shift to fixed night shift work and the duration of night-shift exposure ranged from 1 to 25 years. Andersen et al. interviewed Danish nurses who did night-shift work for 24 years and found that they have increased relative risk (1.86) of developing breast cancer. Age of menarche, parity status, and oral contraceptive use were adjusted for confounders but not smoking history [49]. A 2017 US cohort study of NHSII (U.S.-based Nurses’ Health Study II) of 116,430 women showed that nurses who did at least three nights per month have increased relative risk of breast cancer and this study had adjusted multiple confounders as well [50]. In a 2012 Swedish cohort study of 4053 women who did at least one night-shift work and followed for 12 years, it was shown that there is increased relative risk of developing breast cancer. This study did not specify the type of night-shift work that the participants did [51]. Large US cohort studies by Schernhammer et al. in 2001 using data from the Nurses Health Study demonstrated a moderate risk increase in nurses who worked 1-14 years (RR 1.08) or 15-29 years on rotating night shifts (RR 1.08). The risk was further increased among women who worked 30 or more years on the night shift [8]. A similar cohort study by the same author demonstrated the same result for female nurses who have done at least 20 years of night work [9]. Wegrzyn et al. analyzed the two previously studied cohort of nurses who did night shift from Nurses’ Health Study I and II and found that women with 20 years or more of cumulative rotating night-shift work exposure had a marginally significant higher risk of breast cancer with a hazard ratio of 1.40 and strongest associations were seen in women who did night-shift work as young adults. Interestingly, this study also showed lower mammography screening with increasing shift work. Women who did shift work also tend to have higher body weight and tend to be current smokers but have lower alcohol consumption [52]. 

In contrast, there are significant studies that contributed to the null hypothesis. Travis et al. utilized data from Million Women Study, EPIC-Oxford, and UK Biobank participants and found that there is little to no evidence of night-shift work increasing the risk of breast cancer [53]. A Danish cohort study did not demonstrate the short-term effect of breast cancer risk from night-shift work [54]. Sweeney et al. utilized data from the Sister Study cohort which included participants who have a sister with breast cancer but are cancer free themselves and found that there is little evidence of night-shift work increasing the risk of breast cancer. Contrasting results were seen in short-term night-shift work [55]. Lack of association was also seen in the Chinese [56] and Dutch studies [57].

Night Shift and Other Malignancies

Evidence pertaining to night-shift work to other cancer is controversial. A Canadian population-based case-control study investigating association between night-shift work and prostate cancer showed that no association was found between overall prostate cancer and night-shift work metrics, including ever exposure, duration, intensity, cumulative exposure, rotating shifts, and early-morning shifts [58]. A 2020 systematic review and meta-analysis by Rivera-Izquierdo et al. demonstrated no association between night-shift work and prostate cancer; however, it is noted that most studies that showed positive association tend to be low in quality and have publication bias [17]. Haghayegh et al. utilized data from NSH I and II to investigate the association between bladder cancer risk and night-shift work and found no association [16]. Schernhammer et al. analyzed the association between night-shift work and lung cancer and found that there is a 28% increased risk among women who did at least 15 years of night-shift work and the strongest association was seen with small cell carcinoma, although of note the results could be confounded by smoking [15]. A systematic review by Schwarz et al. analyzing the association between gynecological cancer and night-shift work demonstrated that there is no clear correlation between night-shift work and risk of developing endometrial, ovarian, and cervical cancer [59]. A 2013 study examining the risk of dying from pancreatic cancer in 22,224 Japanese men demonstrated no significant association between night-shift work and pancreatic cancer mortality [60]. Another Japanese study by Japan Collaborative Cohort Study (JACC) in 2021 showed that rotating night-shift work is associated with increased risk of esophageal cancer in men, but fixed night-shift work and rotating night-shift work were not associated with other types of cancer in either sex [18].

Different Types of Night-Shift Patterns and Other Non-healthcare Night-Shift Workers

There are limited studies that compare different types of night-shift work. An observational study of women in the Danish military included both nurses and non-nurses; while it affirmed previous studies’ conclusions, it did not add any data regarding any difference in those groups [61].

Some available studies did categorize night-shift workers into fixed night shift versus rotating night shift [8,9,52,55,57]; while Koppes et al. showed the strongest association between breast cancer and rotating night-shift work [57], Sweeney et al. did not demonstrate a strong association in either group [55], and Schernhammer et al. did not document comparative analysis between those two groups [8,9]. Available evidence is leaning into cumulative risk with more night shifts performed [8,9,52]. In the 2021 JACC study, the participants were categorized into rotating night-shift workers and fixed night-shift workers; however, the data did not support increased risk of breast cancer with either rotating night-shift work or fixed night-shift work. There is a paucity of data comparing healthcare workers who work the night shift with non-healthcare workers in different types of settings.

Future advances with current evidence in clinical practice

Lack of Evidence Regarding Different Types of Night-Shift Work

Despite having sufficient evidence of possible risk of night-shift work in cancer incidence and relationship to circadian rhythm and melatonin, there is lack of prevention-based studies in this regard. There is evidence that poor sleep quality and quantity in night-shift workers confer increased cancer risk [62,63]; therefore, improving sleep quality and ensuring sufficient sleep duration are of extreme importance in night-shift workers. It is also necessary to conduct diverse studies of night-shift workers since the stress levels may be different, e.g. conducting studies on factory workers, healthcare workers in different types of settings, such as nurses in floor setting or critical care setting, and physicians who work solely at nights such as nocturnists. More studies are also needed to analyze any difference in risk between rotating and fixed night-shift work.

Melatonin as Cancer Prevention and Minimizing Circadian Rhythm Disruption During Night Shift 

Melatonin has been used in various clinical trials of cancer treatment including breast cancer and prostate cancer as effective adjunctive therapies [64]. Melatonin has been studied extensively in breast cancer possibly due to its role in estrogen mechanism. Melatonin can induce apoptosis in cancer-ridden cells, has anti-estrogenic effects, and inhibits angiogenesis [65]. In current clinical practices, despite its promises, melatonin’s administration as cancer treatment still needs further studies before it can be implemented in clinical practices as researchers are still investigating the optimal dose and administration as cancer therapy. Moreover, some conflicting results do exist [66]. There has not been a study dedicated to studying melatonin as preventive treatment for night-shift workers. With increasing evidence of night-shift workers and their cancer risk, it is imperative to conduct this type of experiment. Since there is evidence in animals that artificial lights at night can increase cancer risk [2,23], it might be imperative to conduct studies examining artificial light at night with cancer risk in night-shift workers and if implementing devices such as blue light-blocking glasses may be beneficial [67].

*Adopting Healthier Lifestyles in Night-Shift Workers*

Studies have shown that night shift workers tend to have unhealthier lifestyle choices, but it remains unclear whether this contributes to the risk of cancer. Adopting a healthier lifestyle such as increasing physical activity and implementing better nutrition could reduce the risk of developing metabolic disorders in night-shift workers [36], but its role in mitigating cancer risk remains to be proven.

## Conclusions

Most studies that are published focus on the relationship between night-shift work and breast cancer development. There is some evidence in cohort studies that night shift work increases the risk of developing breast cancer. There are much fewer studies that examine the correlation between night-shift work and other types of cancer and the evidence is scarce. Future studies need to be done to investigate the risk of cancer in different age groups. It is unclear if different types of night shift such as fixed versus rotating night shift work have any difference in cancer risk. It is also necessary to conduct studies comparing the risk of developing cancer in different types of night shift workers in both healthcare and non-healthcare fields. Studies included in this manuscript are mostly conducted on healthcare workers who worked night shifts; therefore, the evidence is unable to be applied to the general population and other types of night shift workers such as factory workers and hospitality workers.

The roles of circadian rhythm and melatonin in cancer properties are well known, but the evidence to indicate melatonin as the prevention method is scarce. Future studies to examine holistic prevention methods such as improving sleep quality, nutrition, and exercise and their roles in mitigating cancer risk in night shift workers are also essential.

## References

[1] Rajaratnam SM, Arendt J (2001). Health in a 24-h society. Lancet.

[2] Straif K, Baan R, Grosse Y (2007). Carcinogenicity of shift-work, painting, and fire-fighting. Lancet Oncol.

[3] Hill SM, Frasch T, Xiang S, Yuan L, Duplessis T, Mao L (2009). Molecular mechanisms of melatonin anticancer effects. Integr Cancer Ther.

[4] Straif K, Silverstein M (1997). Comparison of occupational safety and health administration standards and German Berufsgenossenschaften guidelines for preventive occupational health examinations. Am J Ind Med.

[5] Hill SM, Belancio VP, Dauchy RT (2015). Melatonin: an inhibitor of breast cancer. Endocr Relat Cancer.

[6] Bracci M, Manzella N, Copertaro A (2014). Rotating-shift nurses after a day off: peripheral clock gene expression, urinary melatonin, and serum 17-β-estradiol levels. Scand J Work Environ Health.

[7] Schernhammer ES, Rosner B, Willett WC, Laden F, Colditz GA, Hankinson SE (2004). Epidemiology of urinary melatonin in women and its relation to other hormones and night work. Cancer Epidemiol Biomarkers Prev.

[8] Schernhammer ES, Laden F, Speizer FE, Willett WC, Hunter DJ, Kawachi I, Colditz GA (2001). Rotating night shifts and risk of breast cancer in women participating in the nurses' health study. J Natl Cancer Inst.

[9] Schernhammer ES, Kroenke CH, Laden F, Hankinson SE (2006). Night work and risk of breast cancer. Epidemiology.

[10] Wei T, Li C, Heng Y (2020). Association between night-shift work and level of melatonin: systematic review and meta-analysis. Sleep Med.

[11] Booker LA, Magee M, Rajaratnam SM, Sletten TL, Howard ME (2018). Individual vulnerability to insomnia, excessive sleepiness and shift work disorder amongst healthcare shift workers. A systematic review. Sleep Med Rev.

[12] Wei F, Chen W, Lin X (2022). Night-shift work, breast cancer incidence, and all-cause mortality: an updated meta-analysis of prospective cohort studies. Sleep Breath.

[13] Lin X, Chen W, Wei F, Ying M, Wei W, Xie X (2015). Night-shift work increases morbidity of breast cancer and all-cause mortality: a meta-analysis of 16 prospective cohort studies. Sleep Med.

[14] Ijaz S, Verbeek J, Seidler A (2013). Night-shift work and breast cancer--a systematic review and meta-analysis. Scand J Work Environ Health.

[15] Schernhammer ES, Feskanich D, Liang G, Han J (2013). Rotating night-shift work and lung cancer risk among female nurses in the United States. Am J Epidemiol.

[16] Haghayegh S, Liu Y, Zhang Y (2023). Rotating night shift work and bladder cancer risk in women: results of two prospective cohort studies. Int J Environ Res Public Health.

[17] Rivera-Izquierdo M, Martínez-Ruiz V, Castillo-Ruiz EM, Manzaneda-Navío M, Pérez-Gómez B, Jiménez-Moleón JJ (2020). Shift work and prostate cancer: an updated systematic review and meta-analysis. Int J Environ Res Public Health.

[18] Arafa A, Eshak ES, Iso H, Muraki I, Tamakoshi A (2021). Night work, rotating shift work, and the risk of cancer in Japanese men and women: The JACC Study. J Epidemiol.

[19] Brzezinski A (1997). Melatonin in humans. N Engl J Med.

[20] Fu L, Lee CC (2003). The circadian clock: pacemaker and tumour suppressor. Nat Rev Cancer.

[21] Lee S, Donehower LA, Herron AJ, Moore DD, Fu L (2010). Disrupting circadian homeostasis of sympathetic signaling promotes tumor development in mice. PLoS One.

[22] Wood PA, Yang X, Taber A (2008). Period 2 mutation accelerates ApcMin/+ tumorigenesis. Mol Cancer Res.

[23] Hu L, Li G, Shu Y, Hou X, Yang L, Jin Y (2022). Circadian dysregulation induces alterations of visceral sensitivity and the gut microbiota in Light/Dark phase shift mice. Front Microbiol.

[24] Bollati V, Baccarelli A, Sartori S, Tarantini L, Motta V, Rota F, Costa G (2010). Epigenetic effects of shiftwork on blood DNA methylation. Chronobiol Int.

[25] Talib WH (2018). Melatonin and cancer hallmarks. Molecules.

[26] Acuña-Castroviejo D, Escames G, Venegas C (2014). Extrapineal melatonin: sources, regulation, and potential functions. Cell Mol Life Sci.

[27] Bonnefont-Rousselot D, Collin F (2010). Melatonin: action as antioxidant and potential applications in human disease and aging. Toxicology.

[28] Miller SC, Pandi-Perumal SR, Esquifino AI, Cardinali DP, Maestroni GJ (2006). The role of melatonin in immuno-enhancement: potential application in cancer. Int J Exp Pathol.

[29] Mehrzadi S, Pourhanifeh MH, Mirzaei A, Moradian F, Hosseinzadeh A (2021). An updated review of mechanistic potentials of melatonin against cancer: pivotal roles in angiogenesis, apoptosis, autophagy, endoplasmic reticulum stress and oxidative stress. Cancer Cell Int.

[30] Poon AM, Liu ZM, Pang CS, Brown GM, Pang SF (1994). Evidence for a direct action of melatonin on the immune system. Biol Signals.

[31] Currier NL, Miller SC (2001). Echinacea purpurea and melatonin augment natural-killer cells in leukemic mice and prolong life span. J Altern Complement Med.

[32] Srinivasan V, Spence DW, Pandi-Perumal SR, Trakht I, Cardinali DP (2008). Therapeutic actions of melatonin in cancer: possible mechanisms. Integr Cancer Ther.

[33] Sanchez-Barcelo EJ, Mediavilla MD, Alonso-Gonzalez C, Reiter RJ (2012). Melatonin uses in oncology: breast cancer prevention and reduction of the side effects of chemotherapy and radiation. Expert Opin Investig Drugs.

[34] Nooshinfar E, Safaroghli-Azar A, Bashash D, Akbari ME (2017). Melatonin, an inhibitory agent in breast cancer. Breast Cancer.

[35] Konopleva M, Martinelli G, Daver N (2020). MDM2 inhibition: an important step forward in cancer therapy. Leukemia.

[36] Shan Z, Li Y, Zong G (2018). Rotating night shift work and adherence to unhealthy lifestyle in predicting risk of type 2 diabetes: results from two large US cohorts of female nurses. BMJ.

[37] Pepłońska B, Nowak P, Trafalska E (2019). The association between night shift work and nutrition patterns among nurses: a literature review. Med Pr.

[38] Pepłońska B, Burdelak W, Krysicka J (2014). Night shift work and modifiable lifestyle factors. Int J Occup Med Environ Health.

[39] Borek C (2004). Dietary antioxidants and human cancer. Integr Cancer Ther.

[40] Alegría-Torres JA, Baccarelli A, Bollati V (2011). Epigenetics and lifestyle. Epigenomics.

[41] Buchvold HV, Pallesen S, Øyane NM, Bjorvatn B (2015). Associations between night work and BMI, alcohol, smoking, caffeine and exercise--a cross-sectional study. BMC Public Health.

[42] Zhang FF, Cardarelli R, Carroll J (2011). Physical activity and global genomic DNA methylation in a cancer-free population. Epigenetics.

[43] Schulz WA, Steinhoff C, Florl AR (2006). Methylation of endogenous human retroelements in health and disease. Curr Top Microbiol Immunol.

[44] Puttonen S, Härmä M, Hublin C (2010). Shift work and cardiovascular disease — pathways from circadian stress to morbidity. Scand J Work Environ Health.

[45] Robinson CF, Sullivan PA, Li J, Walker JT (2011). Occupational lung cancer in US women, 1984-1998. Am J Ind Med.

[46] Cho YS, Kim HR, Myong JP, Kim HW (2013). Association between work conditions and smoking in South Korea. Saf Health Work.

[47] Knutsson A, Nilsson T (1998). Tobacco use and exposure to environmental tobacco smoke in relation to certain work characteristics. Scand J Soc Med.

[48] (2021). NTP Cancer Hazard Assessment Report on Night Shift Work and Light at Night.

[49] Andersen ZJ, Jørgensen JT, Elsborg L (2018). Long-term exposure to road traffic noise and incidence of breast cancer: a cohort study. Breast Cancer Res.

[50] James P, Bertrand KA, Hart JE, Schernhammer ES, Tamimi RM, Laden F (2017). Outdoor light at night and breast cancer incidence in the Nurses' Health Study II. Environ Health Perspect.

[51] Knutsson A, Alfredsson L, Karlsson B, Åkerstedt T, Fransson EI, Westerholm P, Westerlund H (2013). Breast cancer among shift workers: results of the WOLF longitudinal cohort study. Scand J Work Environ Health.

[52] Wegrzyn LR, Tamimi RM, Rosner BA (2017). Rotating night-shift work and the risk of breast cancer in the Nurses' Health Studies. Am J Epidemiol.

[53] Travis RC, Balkwill A, Fensom GK (2016). Night shift work and breast cancer incidence: three prospective studies and meta-analysis of published studies. J Natl Cancer Inst.

[54] Vistisen HT, Garde AH, Frydenberg M (2017). Short-term effects of night shift work on breast cancer risk: a cohort study of payroll data. Scand J Work Environ Health.

[55] Sweeney MR, Sandler DP, Niehoff NM, White AJ (2020). Shift work and working at night in relation to breast cancer incidence. Cancer Epidemiol Biomarkers Prev.

[56] Pronk A, Ji BT, Shu XO (2010). Night-shift work and breast cancer risk in a cohort of Chinese women. Am J Epidemiol.

[57] Koppes LL, Geuskens GA, Pronk A, Vermeulen RC, de Vroome EM (2014). Night work and breast cancer risk in a general population prospective cohort study in The Netherlands. Eur J Epidemiol.

[58] Barul C, Richard H, Parent ME (2019). Night-shift work and risk of prostate cancer: results from a Canadian case-control study, the prostate cancer and environment study. Am J Epidemiol.

[59] Schwarz C, Pedraza-Flechas AM, Lope V, Pastor-Barriuso R, Pollan M, Perez-Gomez B (2018). Gynaecological cancer and night shift work: a systematic review. Maturitas.

[60] Lin Y, Ueda J, Yagyu K, Kurosawa M, Tamakoshi A, Kikuchi S (2013). A prospective cohort study of shift work and the risk of death from pancreatic cancer in Japanese men. Cancer Causes Control.

[61] Hansen J, Lassen CF (2012). Nested case-control study of night shift work and breast cancer risk among women in the Danish military. Occup Environ Med.

[62] Cordina-Duverger E, Uchai S, Tvardik N (2022). Sleep traits, night shift work and lung cancer risk among women: results from a population-based case-control study in France (The WELCA Study). Int J Environ Res Public Health.

[63] White AJ, Weinberg CR, Park YM, D'Aloisio AA, Vogtmann E, Nichols HB, Sandler DP (2017). Sleep characteristics, light at night and breast cancer risk in a prospective cohort. Int J Cancer.

[64] Talib WH, Alsayed AR, Abuawad A, Daoud S, Mahmod AI (2021). Melatonin in cancer treatment: current knowledge and future opportunities. Molecules.

[65] Li Y, Li S, Zhou Y, Meng X, Zhang JJ, Xu DP, Li HB (2017). Melatonin for the prevention and treatment of cancer. Oncotarget.

[66] Wang L, Wang C, Choi WS (2022). Use of melatonin in cancer treatment: where are we?. Int J Mol Sci.

[67] Esaki Y, Takeuchi I, Tsuboi S, Fujita K, Iwata N, Kitajima T (2020). A double-blind, randomized, placebo-controlled trial of adjunctive blue-blocking glasses for the treatment of sleep and circadian rhythm in patients with bipolar disorder. Bipolar Disord.

